# Supplementary material for: Bistable nerve conduction
Source: Biophys J. 2022 Aug 12;121(18):3499–507. doi: 10.1016/j.bpj.2022.08.006 (PMC9515125; doi:10.1016/j.bpj.2022.08.006)
Supplement: Document S1. Figures S1–S5 [file mmc1.pdf]

Biophysical Journal, Volume *121*

Supplemental information

Bistable nerve conduction

Zhaoyang Zhang and Zhilin Qu

Supplemental Information

A. Supplemental Methods

1. Assigned intervals for random parameter drawing

To evaluate the robustness of the two mechanisms, we carried out a large number of simulations with randomly drawn parameter sets. In these simulations, besides drawing the maximum conductance and time constants randomly, we also randomly shifted the kinetics of the ionic currents to investigate the effects of their activation thresholds on conduction. To shift the I_{Na} kinetics, we replaced V for α_m, β_m, α_h, and β_h by ($V+V_{Na,shift}$). In the control, the half-activation potential for I_{Na} is $V_{Na,1/2} \approx -35\ mV$ (at which m_∞=0.5). After the shift, the half-activation potential becomes,

$$V_{Na,1/2} = -35 - V_{Na,shift} \qquad \text{(S1)}$$

Similarly, we shifted the I_{Ca} kinetics by replacing V for d_∞ and f_∞ with ($V+V_{Ca,shift}$). The half-activation voltage for I_{Ca} at control is $V_{Ca,1/2} = -14\ mV$ (at which d_∞=0.5), and after the shift, it becomes,

$$V_{Ca,1/2} = -14 - V_{Ca,shift} \qquad \text{(S2)}$$

We also shifted the I_K kinetics by replacing V for α_n and β_n with ($V+V_{K,shift}$).

The randomly drawn parameter sets used for the simulations for Fig.3, Fig.4, and Fig.S2 were uniformly drawn from the following pre-assigned intervals: $G_{Na}\in[50,200]$, $G_K\in[20,60]$, $G_{Ca}\in[0,10]$, $V_{Na,shift}\in[-75,25]$, $V_{Ca,shift}\in[-50,50]$, $V_{K,shift}\in[-40,60]$, $\gamma_m\in[0.1,0.5]$, $\gamma_d\in[1,8]$, $\gamma_n\in[0.5,10]$, $k_f\in[8,2]$. The fixed parameters were the same as for control. Therefore, based on Eqs.S1 and S2 and the assigned intervals for $V_{Na,shift}$ and $V_{Ca,shift}$, the range for $V_{Na,1/2}$ is [-60, 40] mV and that for $V_{Ca,1/2}$ is [-64, 36] mV.

2. Theoretical model and analysis of bistable conduction

To understand the mechanism of bistable conduction mediated by Na^+ channel, we investigated the following simple cable equation:

$$\frac{\partial V}{\partial t} = \frac{\partial^2 V}{\partial x^2} + \sigma_{Na} m_\infty(V) h - G_L(V - E_L) \qquad \text{(S3)}$$

$$\frac{\partial h}{\partial t} = \frac{h_\infty(V) - h}{\tau_h} \qquad \text{(S4)}$$

For simplicity and analytical treatment, we set the diffusion constant D=1, and set $\sigma_{Na} = G_{Na}(E_{Na} - V_C)$ to be single parameter. We also assume τ_h is a constant independent of V. $m_\infty(V)$ and $h_\infty(V)$ are Heaviside functions of V as follows:

$$m_\infty(V) = \begin{cases} 0, & V < V_m \\ 1, & V \geq V_m \end{cases} \qquad \text{(S5)}$$

and

$$h_\infty(V) = \begin{cases} 1, & V < V_h \\ h_0, & V \geq V_h \end{cases} \qquad \text{(S6)}$$

We seek a travelling wave solution V(x, t) with speed c in an infinite spatial domain: $x \in (-\infty, +\infty)$, which becomes a stationary front in the moving coordinate system (see Fig.S4) $\varepsilon = x - ct$, i.e.,

$$V(x,t) = U(x-ct) = U(\varepsilon) \qquad \text{(S7)}$$

By substituting Eq.S7 into Eq.S3 and Eq.S4, one obtains the follow set of differential equations:

$$0 = U_{\varepsilon\varepsilon} + cU_{\varepsilon} + \sigma_{Na} m_{\infty} h - G_L(U - E_L) \qquad \text{(S8)}$$

$$h_{\varepsilon} = \frac{h_{\infty}(U) - h}{-c\tau_h} \qquad \text{(S9)}$$

in which $U_{\varepsilon} = \frac{dU}{d\varepsilon}$ and $U_{\varepsilon\varepsilon} = \frac{dU_{\varepsilon}}{d\varepsilon}$. The solution U(ε) satisfies the following boundary conditions: 1) $U_{\varepsilon} = U_{\varepsilon\varepsilon} = 0$ at $\varepsilon \to \pm\infty$; and 2) We assume that $m_{\infty}(V)$ changes from 1 to 0 at $\varepsilon = 0$, then $U(0) = V_m$ and $U_{\varepsilon}|_{\varepsilon=0^-} = U_{\varepsilon}|_{\varepsilon=0^+}$. Therefore, at the boundaries, U(ε) satisfies:

$$U(\varepsilon) = \begin{cases} U_0, & \varepsilon \to -\infty \\ V_m, & \varepsilon = 0 \\ E_L, & \varepsilon \to +\infty \end{cases} \qquad \text{(S10)}$$

where $U_0 = \frac{\sigma_{Na} h_0 + G_L E_L}{G_L}$. Now the problem is to solve the linear differential equations in the subdomains of $\varepsilon < 0$ and $\varepsilon > 0$.

(i) $\varepsilon > 0$

Since $m_{\infty} = 0$ when $\varepsilon > 0$, then Eq.S8 becomes:

$$0 = U_{\varepsilon\varepsilon} + cU_{\varepsilon} - G_L(U - E_L) \qquad \text{(S11)}$$

which has the following form of solution:

$$U(\varepsilon) = Ae^{\lambda_- \varepsilon} + E_L \qquad \text{(S12)}$$

where $\lambda_- = \frac{-c - \sqrt{c^2 + 4G_L}}{2}$ is a solution of the characteristic equation $\lambda^2 + c\lambda - G_L(U - E_L) = 0$. Since $\lambda_- < 0$, $U(\varepsilon) = E_L$ at $\varepsilon \to +\infty$ is satisfied. Since $U(\varepsilon) = V_m$ at $\varepsilon = 0$, one obtains $A = V_m - E_L$. The solution of Eq.S9 in the whole spatial domain is:

$$h(\varepsilon) = \begin{cases} h_0 + (1 - h_0)e^{\frac{\varepsilon - \varepsilon_0}{c\tau_h}}, & \varepsilon < \varepsilon_0 \\ 1, & \varepsilon \geq \varepsilon_0 \end{cases} \qquad \text{(S13)}$$

in which ε_0 is determined by $(V_m - E_L)e^{\lambda_- \varepsilon_0} + E_L = V_h$, which leads to

$$\varepsilon_0 = \frac{ln\left(\frac{V_h - E_L}{V_m - E_L}\right)}{\lambda_-} \qquad \text{(S14)}$$

(ii) $\varepsilon < 0$

Since $m_{\infty} = 1$ when $\varepsilon < 0$, using Eq.S13 for h, then Eq.S8 becomes

$$0 = U_{\varepsilon\varepsilon} + cU_{\varepsilon} + \sigma_{Na}\left[h_0 + (1 - h_0)e^{\frac{\varepsilon - \varepsilon_0}{c\tau_h}}\right] - G_L(U - E_L) \qquad \text{(S15)}$$

Eq.S15 exhibits the following form of solution:

$$U(\varepsilon) = U_0 + Be^{\lambda_+\varepsilon} + \beta e^{\frac{\varepsilon}{c\tau_h}} \qquad \text{(S16)}$$

where $\lambda_+ = \frac{-c+\sqrt{c^2+4G_L}}{2}$ is a solution of the characteristic equation $\lambda^2 + c\lambda - G_L(U - E_L) = 0$. Since $\lambda_+ > 0$, $U(\varepsilon) = U_0$ at $\varepsilon \to -\infty$ is satisfied. Since $U(\varepsilon) = V_m$ at $\varepsilon = 0$, one obtains $B = V_m - \beta - U_0$. Inserting Eq.S16 into Eq.S15, one obtains the following equation for β:

$$\frac{\beta}{(c\tau_h)^2} + \frac{c\beta}{c\tau_h} + \sigma_{Na}(1 - h_0)e^{\frac{-\varepsilon_0}{c\tau_h}} - \beta G_L = 0 \qquad \text{(S17)}$$

which gives rise to $\beta = \frac{\sigma_{Na}(1-h_0)e^{\frac{-\varepsilon_0}{c\tau_h}}}{G_L - \frac{1}{(c\tau_h)^2} - \frac{1}{\tau_h}}$.

Finally, applying the constrain that U is smooth at $\varepsilon = 0$, i.e., $U_\varepsilon|_{\varepsilon=0^-} = U_\varepsilon|_{\varepsilon=0^+}$, one has from Eq.S12 and Eq.S16 the following relation:

$$A\lambda_- = B\lambda_+ + \frac{\beta}{c\tau_h} \qquad \text{(S18)}$$

which leads to

$$-(V_m - E_L)\sqrt{c^2 + 4G_L} + \sigma_{Na}[(\beta + \frac{h_0}{G_L})\lambda_+ - \frac{\beta}{\tau_h c}] = 0 \qquad \text{(S19)}$$

Therefore, once the parameters are given, one can solve Eq.S19 to obtain the conduction speed c as a function of anyone of the parameters, which is discussed in the main text. Fig.S4 plots the two stable and the unstable traveling wave solutions of Eq.S12 and Eq.S16 with the c values calculated from Eq.S19.

B. Supplemental Figures

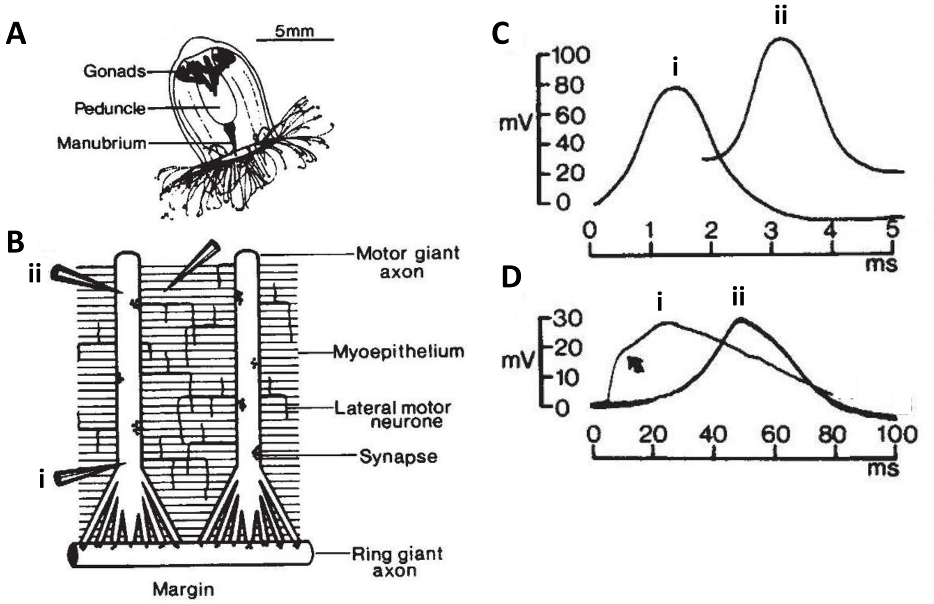

Figure S1. Action potential conduction in giant axon of *Aglantha digitale*. A. Schematic drawing of *Aglantha digitale*. **B**. Recording sites (i and ii) in the giant axon. **C**. Recorded action potentials during an escape swim, which are I_{Na}-mediated high amplitude and short duration excitations. **D**. Recorded action potentials during slow swim, which are I_{Ca}-mediated low amplitude and long duration excitations. This figure is modified from Mackie and Meech (1), see the original paper for a detailed description.

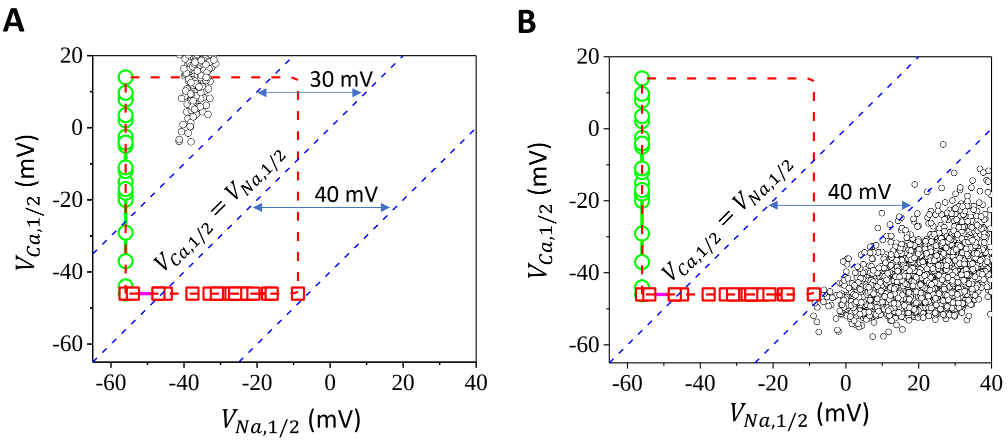

Figure S2. **Robustness of bistable conduction. A**. Parameter sets (black) exhibiting stimulus-dependent fast and slow conduction after I_{Ca} was removed from the model using the parameter sets in Fig.4D. The data points in the lower-right group are gone but there are data points in the upper-left group. This indicates that the upper-left group in Fig.4D may still undergo the bistable conduction even though the parameter sets were filtered using the dual-threshold criterion. **B**. Parameter sets (black) exhibiting stimulus-dependent fast and slow conduction after changing γ_h=0.35 to γ_h=1. Unlike Figs. 4 C or D, the data points in this panel were not filtered with the threshold criterion.

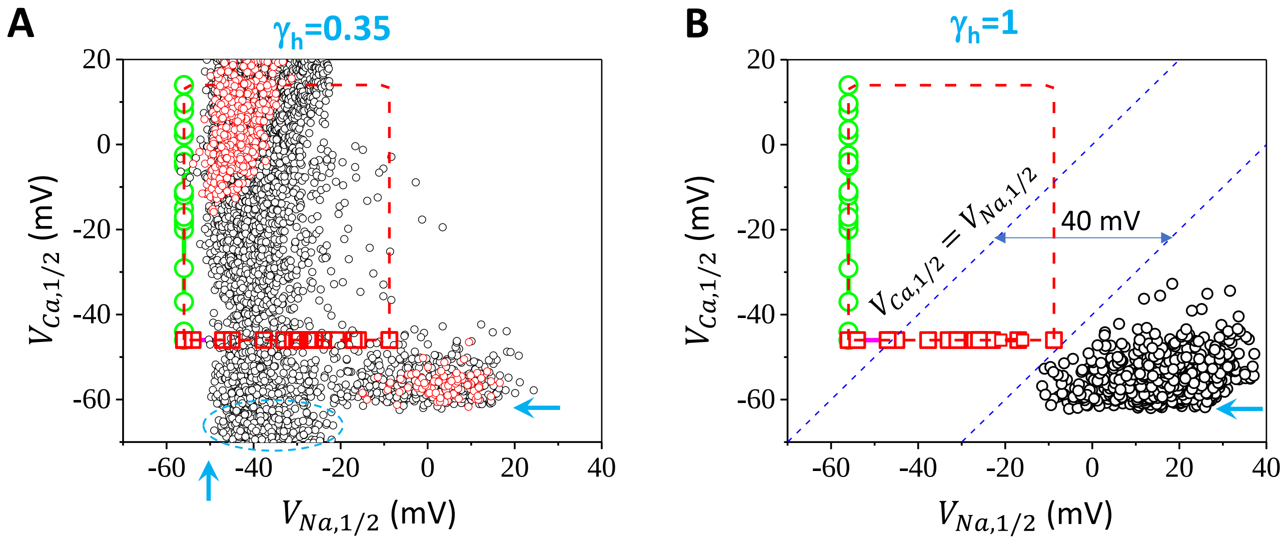

Figure S3. **Effects of lowering the resting potential**. Data sets exhibiting fast and slow conduction after changing the resting potential from -65 mV to -75 mV by changing E_L=-65 mV to E_L=-75 mV. **A**. γ_h=0.35. Red dots are the parameter sets filtered with the dual-threshold criterion. Black dots are the parameter sets filtered with the single-threshold criterion. **B**. γ_h=1. Simulation protocols and parameters are the same as for Fig.4 in the main text except the mentioned changes. The lowering of the resting potential caused two changes: down-shift of the $V_{Ca,1/2}$ boundaries (marked by horizontal arrows, ~10 mV lower comparing to Fig.4) and left-shift of the $V_{Na,1/2}$ boundary in A (marked by the vertical arrow, ~10 mV left-shifted comparing to Fig.4C). In other words, shifting the resting from -65 mV to -75 mV results in a ~10 mV shift in these boundaries. Beyond these boundaries, spontaneous oscillations occur and thus no conduction in the cable due to the low activation thresholds. The data points in the region marked by the dashed circle are the bistable conduction caused by I_{Na} alone in the absence of or under weak I_{Ca} so that the lower boundary becomes lower than the oscillation boundary caused by I_{Ca}.

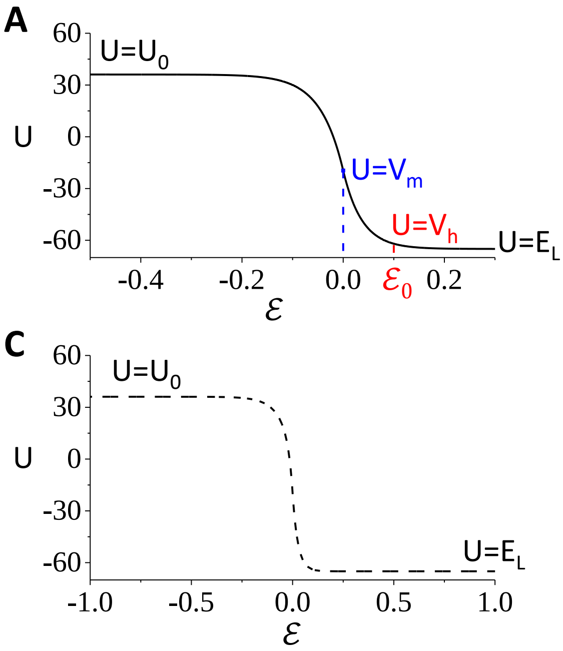

Figure S4. **Stable and unstable wavefronts in the moving coordinate from the simplified model**. **A**. The stable slow wave front of a bistable conduction. U=V_m when ε=0, which gives rise to m_∞=1 when ε<0 and m_∞=0 when ε>0. U=V_h when ε=ε_0, which gives rise to h_∞=h_0 when ε<ε_0 and h_∞=1 when ε>ε_0. **B**. The stable fast wave front of the same bistable conduction as in A. **C**. The unstable wave front of the same bistable conduction as in A and B. The traveling wave solutions in A-C were plots of Eq.S12 and Eq.S16 using c values calculated in Eq.S19 with the following parameter set: $\tau_h = 1.6$, $V_m = -19.5$ mV, $V_h = -55.5$ mV, $h_0 = 0.075$, $G_L = 0.3$, $E_L = -65$ mV. $G_{Na} = 190$ mS/cm^2. $\sigma_{Na} = 2.1G_{Na}$.

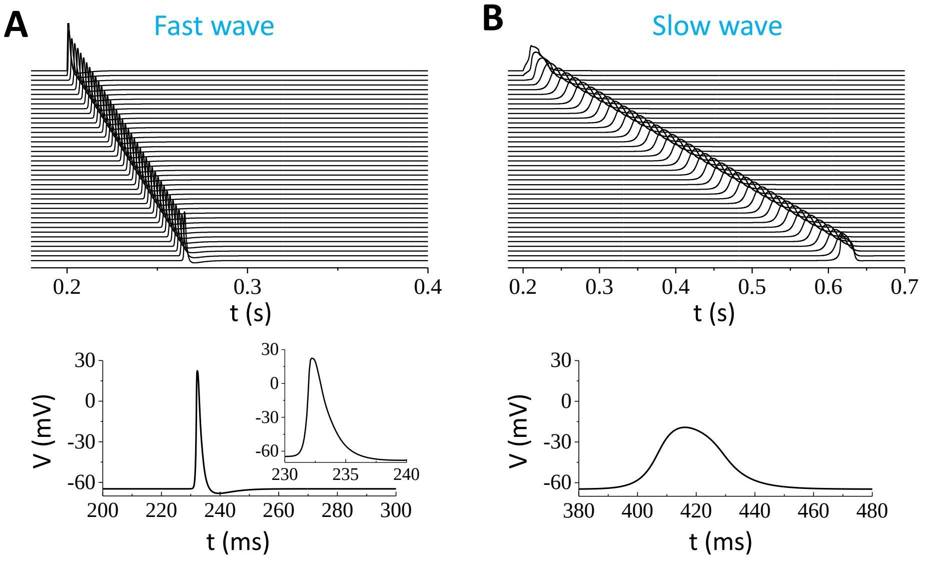

Figure S5. **Bistable conduction in the presence of A-type current**. Simulations are carried out the same way as for Figs.1 A and B in the main text except that an A-type current is added to the model. The formulation of I_A is taken from Medlock et al (2) with the following parameter changes: $p = 1,\ \Phi_m = -10,\ k_m = 1$ $\Phi_h = -30$, and $\tau_m = 3$.

References

1. Mackie, G. O., and R. W. Meech. 1985. Separate sodium and calcium spikes in the same axon. Nature 313(6005):791-793.
2. Medlock, L., L. Shute, M. Fry, D. Standage, and A. V. Ferguson. 2018. Ionic mechanisms underlying tonic and burst firing behavior in subfornical organ neurons: a combined experimental and modeling study. J Neurophysiol 120(5):2269-2281.
