# Supplementary material for: Bistable nerve conduction
Source: Biophys J. 2022 Aug 12;121(18):3499–507. doi: 10.1016/j.bpj.2022.08.006 (PMC9515125; doi:10.1016/j.bpj.2022.08.006)
Supplement: Document S2. Article plus supporting material [file mmc2.pdf]

Biophysical Journal
Article

Biophysical Society

Bistable nerve conduction

Zhaoyang Zhang[1] and Zhilin Qu[1,2,*]
[1]Department of Medicine, David Geffen School of Medicine, University of California, Los Angeles, Los Angeles, California and [2]Department of Computational Medicine, David Geffen School of Medicine, University of California, Los Angeles, Los Angeles, California

ABSTRACT It has been demonstrated experimentally that slow and fast conduction waves with distinct conduction velocities can occur in the same nerve system depending on the strength or the form of the stimulus, which give rise to two modes of nerve functions. However, the mechanisms remain to be elucidated. In this study, we use computer simulations of the cable equation with modified Hodgkin-Huxley kinetics and analytical solutions of a simplified model to show that stimulus-dependent slow and fast waves recapitulating the experimental observations can occur in the cable, which are the two stable conduction states of a bistable conduction behavior. The bistable conduction is caused by a positive feedback loop of the wavefront upstroke speed, mediated by the sodium channel inactivation properties. Although the occurrence of bistable conduction only requires the presence of the sodium current, adding a calcium current to the model further promotes bistable conduction by potentiating the slow wave. We also show that the bistable conduction is robust, occurring for sodium and calcium activation thresholds well within the experimentally determined ones of the known sodium and calcium channel families. Since bistable conduction can occur in the cable equation of Hodgkin-Huxley kinetics with a single inward current, i.e., the sodium current, it can be a generic mechanism applicable to stimulus-dependent fast and slow conduction not only in the nerve systems but also in other electrically excitable systems, such as cardiac muscles.

SIGNIFICANCE Stimulus-dependent fast and slow conduction waves in the same nerve system have been shown experimentally, which give rise to two modes of nerve functions. We use computer simulations to show that the fast and slow waves are a bistable conduction caused by a positive feedback loop of the wavefront upstroke speed, mediated by the sodium channel inactivation properties, which is further potentiated by the presence of the calcium current. The mechanism is robust with respect to the experimentally determined activation thresholds of the known sodium and calcium channel families. The theoretical insights provide a generic mechanism for stimulus-dependent fast and slow conduction in the nerve systems, which may be also applicable to other electrically excitable tissues, such as cardiac muscles.

INTRODUCTION

The major function of the nerve system is to transmit information via electrical excitation and conduction for the body or parts of the body to respond to environmental changes. The response time is determined by the conduction velocity (CV) of the nerve cable. It is well established that CV is determined by the membrane conductance of the sodium (Na^+) current (I_{Na}) and calcium (Ca^{2+}) current (I_{Ca}), the radius of the cross section of the nerve cable, temperature, and myelination, etc. (1–4). Namely, a larger Na^+ or Ca^{2+} current conductance gives rise to a steeper upstroke of the action potential (or a faster depolarization) to result in a larger CV. A larger nerve cable or myelination effectively enhances the diffusive coupling to give rise to a larger CV. Temperature affects both the ionic currents and the coupling to affect conduction. CV in different nerve fibers or species spans over a wide range, differing in orders of magnitude (1). These conduction properties can be well described by the cable equation with the Hodgkin-Huxley (HH) model (5–10).

Besides the regular conduction properties, an interesting conduction behavior was observed experimentally, i.e., stimulus-dependent fast and slow conduction waves occur in the same nerve fiber. For example, *Aglantha digitale*, a species of jellyfish, has two modes of swimming—a slow swimming for fishing and a fast swimming for escaping away from predators (11,12). In an experimental study (13), Mackie and Meech showed that the slow and fast swimming modes were caused by a slow conduction (~0.3 m/s) and a fast conduction (~1.4 m/s) in the same motor giant axon, respectively. The slow conduction is a

Submitted April 29, 2022, and accepted for publication August 9, 2022.
*Correspondence: zqu@mednet.ucla.edu
Editor: Arthur Sherman.
https://doi.org/10.1016/j.bpj.2022.08.006
© 2022 Biophysical Society.
This is an open access article under the CC BY license (http://creativecommons.org/licenses/by/4.0/).

low-amplitude and long-duration wave mediated by I_{Ca} and the fast conduction is a high-amplitude and short-duration wave mediated by I_{Na} (Fig. S1). The fast wave was induced by an external stimulus and the slow wave was spontaneous and endogenous. This same conduction dynamics was also shown in experiments of conduction in cockroach giant axons by Hochner and Spira (14), who demonstrated that two distinct conduction waves (0.1–0.6 and 3–6 m/s) occurred in the same axon treated with ethanol. Differing from *Aglantha digitale*, the slow conduction in the cockroach giant axon was not mediated by I_{Ca} but still by I_{Na}. Besides conduction in the giant axons of jellyfish and cockroaches, evidence of stimulus-dependent fast and slow conduction has also been shown in experiments of rat visual cortex (15,16), i.e., the CV of the spontaneous waves differ from those of the evoked waves in the same cortex. These experimental observations imply that, besides the regular conduction behavior, a new conduction dynamics can occur in the nerve systems, i.e., the same nerve fiber or tissue can exhibit two stable conduction states depending on the initial conditions. As in *Aglantha digitale*, the two conduction behaviors of the same fiber accomplish two distinct biological functions. However, the underlying mechanism remains to be elucidated.

In this study, we investigate the mechanisms of the stimulus-dependent fast and slow conduction using analytical methods and computer simulations in a cable equation of the HH model (17) with modifications (see Methods). An I_{Ca} formulation is added to the HH model to investigate the role of I_{Ca}. We show that stimulus-dependent fast and slow conduction capturing the experimental observations can occur in the cable with the modified HH model. This is a bistable behavior, namely bistable conduction, emerging during conduction in the cable, caused by a positive feedback loop of the wavefront upstroke speed mediated by the Na^+ channel inactivation properties. The addition of I_{Ca} can further potentiate bistable conduction. Using simulations of randomly selected parameter sets, we show that the bistable conduction mechanism is robust, i.e., the activation thresholds of I_{Na} and I_{Ca} for bistable conduction detected in a wide range of parameters are well within the experimentally determined activation thresholds of the known Na^+ and Ca^{2+} channel families. Since the bistable conduction is mediated by the I_{Na} alone in the HH model, it is likely a generic mechanism applicable to conduction not only in the nerve systems, but also in other electrically excitable media, such as cardiac muscles.

METHODS

Mathematical model

The HH model (17) with modifications is used to simulate action potential conduction in a one-dimensional cable with the following partial differential equation for voltage (V):

$$\frac{\partial V}{\partial t} = -\frac{I_{ion} + I_{stim}}{C_m} + D\frac{\partial^2 V}{\partial x^2}, \tag{1}$$

where $C_m = 1\ \mu F/cm^2$ is the membrane capacitance, and D = 0.0045 cm^2/ms is the diffusion constant. I_{stim} is the stimulus current density and I_{ion} is the total ionic current density consisting of different types of ionic currents, i.e.,

$$I_{ion} = I_{Na} + I_{Ca} + I_K + I_L. \tag{2}$$

In Eq. 2, I_{Na} is the Na^+ current density described by $I_{Na} = G_{Na}m^3h(V - E_{Na})$. I_K is the potassium (K^+) current density described by $I_K = G_K n^4(V - E_K)$. I_L is the leak current density described by $I_L = G_L(V - E_L)$. I_{Ca} is the Ca^{2+} current density described by $I_{Ca} = G_{Ca}d^2f(V - E_{Ca})$. This formulation is adopted from Medlock et al. (18) with the addition of an inactivation gate f. m, h, n, d, and f are gating variables which are described by the following type of differential equations:

$$\frac{dy}{dt} = (y_\infty - y)/\tau_y. \tag{3}$$

In Eq. 3, $y_\infty = \frac{\alpha_y}{\alpha_y+\beta_y}$ and $\tau_y = \frac{1}{\alpha_y+\beta_y}$ in which α and β are rate constants and functions of V.

To observe bistable conduction, we make modifications to the original HH kinetics. We shift the kinetics (see Table 1) to give rise to a resting potential of around −65 mV (the measured resting potential in *Aglantha digitale* is close to this value (13)). Some of the shifts are required for facilitating bistable conduction. Besides the voltage shifts, we also alter the magnitudes of the time constants τ_y by multiplying a prefactor γ_y, i.e.,

$$\tau_y(V) \rightarrow \gamma_y \times \tau_y(V). \tag{4}$$

TABLE 1 Changes of parameters from the original HH model

Parameters	Original HH model	Modified HH model
α_m	$0.1\frac{25 - V}{\exp\left(\frac{25 - V}{10}\right) - 1}$	$0.1\frac{-35 - V}{\exp\left(\frac{-35 - V}{10}\right) - 1}$
β_m	$4\exp\left(\frac{-V}{18}\right)$	$4\exp\left(\frac{-60 - V}{18}\right)$
α_h	$0.07\exp\left(\frac{-V}{20}\right)$	$0.07\exp\left(\frac{-75 - V}{20}\right)$
β_h	$\frac{1}{\exp\left(\frac{30 - V}{10}\right) + 1}$	$\frac{1}{\exp\left(\frac{-45 - V}{10}\right) + 1}$
α_n	$0.01\frac{10 - V}{\exp\left(\frac{10 - V}{10}\right) - 1}$	$0.01\frac{-15 - V}{\exp\left(\frac{-15 - V}{10}\right) - 1}$
β_n	$0.125\exp\left(\frac{-V}{80}\right)$	$0.125\exp\left(\frac{-25 - V}{80}\right)$
E_{Na}	120 mV	55 mV
E_K	−12 mV	−77 mV
E_L	10.6 mV	−65 mV

α_m and β_m are shifted 60 mV toward more negative voltages. α_h and β_h are shifted 75 mV toward more negative voltages. α_n and β_n are shifted 25 mV toward more negative voltages. E_{Na} and E_K are shifted 65 mV toward more negative voltages, and E_L is shifted 75.6 mV. The shifts are to change the resting potential from around zero in the original HH model to −65 mV and to facilitate bistable conduction in the cable.

For d_∞ and f_∞, we use the following formulations: $d_\infty = \frac{1}{1+\exp\left(-\frac{V+14}{k_d}\right)}$ and $f_\infty = \frac{1}{1+\exp\left(\frac{V+44}{k_f}\right)}$. τ_d and τ_f are set as constants independent of V.

The default parameters are set as: $G_K = 36$ mS/cm^2, $G_L = 0.3$ mS/cm^2, $\gamma_m = 0.2$; $\gamma_h = 0.35$, $\gamma_n = 3$; $\tau_d = 3$ ms, $\tau_f = 20$ ms, $k_d = 5.8$, and $k_f = 6$. The values of G_{Na} and G_{Ca} are stated in the figure legends.

We also simulate a nonspatial single-point (or single-cell) model by dropping the diffusion term in Eq. 1, which becomes an ordinary differential equation, i.e.,

$$\frac{dV}{dt} = -\frac{I_{ion} + I_{stim}}{C_m}. \tag{5}$$

Note that after the modifications, the model is still in the excitable regime with a single stable fixed point, i.e., the resting potential. Traveling waves are initiated by I_{stim} which is applied at the beginning of the cable. Details of I_{stim} are stated in the figure legends. Bistable fixed points and oscillatory solutions of Eq. 5 can occur when we use random parameter sets and we exclude these sets from our data shown in Fig. 4.

Numerical methods

A forward Euler method is used for numerical simulation of Eq. 1 with the following discretization:

$$V_i(t+\Delta t) = V_i(t) + \left[-\frac{I_{ion} + I_{stim}}{C_m} + D\frac{[V_{i+1}(t) + V_{i-1}(t) - 2V_i(t)]}{\Delta x^2}\right]\Delta t, \tag{6}$$

with $\Delta x = 0.045$ cm and $\Delta t = 0.005$ ms. No-flux boundary condition is used. The numerical method is stable when $\Delta t < \frac{\Delta x^2}{2D} = 0.225$ ms. The Δt we use is much smaller than this value, ensuring the numerical stability. Eq. 5 is also numerically solved use the Euler method with $\Delta t = 0.005$ ms. The gating variables (Eq. 3) are integrated using the method by Rush and Larsen (19), i.e.,

$$y(t+\Delta t) = y_\infty - [y_\infty - y(t)]e^{-\Delta t/\tau_y} \tag{7}$$

RESULTS

Bistable conduction in the cable equation

To observe bistable conduction in the cable equation with the HH model (17), we modify the model as described in Methods. To examine the role of I_{Ca}, we add an I_{Ca} formulation to the model. Fig. 1, *A* and *B* show a fast wave (1.4 m/s) induced by a strong stimulus and a slow wave (0.21 m/s) induced by a weak stimulus, respectively. The action potential of the fast wave (inset in Fig. 1 *A*) exhibits a steep upstroke, a high amplitude ($\approx$95 mV, from -65 to 30 mV), and a short duration ($\approx$10 ms), while that of the slow wave (inset in Fig. 1 *B*) exhibits a shallow upstroke, a low amplitude ($\approx$45 mV, from -65 to -20 mV), and a long duration ($\approx$40 ms). These features recapitulate well the experimental observations in the jellyfish (see Fig. S1) and cockroach experiments.

To show how the bistable conduction in the cable is affected by I_{Na} and I_{Ca}, we scan the maximum conductance of I_{Na} (G_{Na}) and that of I_{Ca} (G_{Ca}), and plot the CV in color in the two-parameter plane (Fig. 1 *C*). For each parameter set, a strong stimulus and a weak stimulus, as in Fig. 1, *A* and *B*, are applied to elicit conduction waves. The gray region in Fig. 1 *C* is where bistable conduction occurs. The white region is where both stimuli fail to elicit conduction (conduction failure). The colored regions (I, II, and III) are where both stimuli give rise to a single stable conduction with CV color coded. Regions I and II exhibit stable fast conduction mediated by I_{Na}, and region III exhibits stable slow conduction mediated by I_{Ca}. The presence of I_{Ca} promotes bistable conduction; however, I_{Ca} is not required since the bistable conduction occurs in the absence of I_{Ca} ($G_{Ca} = 0$). In the absence of I_{Ca} ($G_{Ca} = 0$), bistable conduction occurs in an intermediate range of G_{Na}, and the fast conduction occurs when G_{Na} is either above or below this range. Therefore, bistable conduction can occur in the HH model mediated by I_{Na} alone without requiring the presence of I_{Ca} or another inward current. In Fig. 1, *D* and *E*, we plot CV versus G_{Na} for $G_{Ca} = 3$ μA/cm^2 and $G_{Ca} = 0$, respectively, showing typical hysteresis, a hallmark of bistability.

Mechanism of bistable conduction

Since bistable conduction can occur in the absence of I_{Ca}, we investigate how it occurs in the cable without I_{Ca}, focusing on the role of I_{Na}. Fig. 2 *A* shows a fast wave induced by a stimulus slightly above the critical strength and a slow wave by a stimulus slightly below the critical strength in the absence of I_{Ca}. In the first 40 ms, the action potentials are almost identical for the two cases, which then bifurcate (as indicated by the *arrow*) into a stable fast wave and a stable slow wave. Since the stimuli are very close to the critical strength, the two waves in the first 40 ms are close to the solution of the unstable conduction. The open circles in Fig. 1 *D* are calculated from the unstable conduction. In the three conduction behaviors, besides the difference in the amplitude and duration of the action potentials, another important difference is the upstroke speed of the wavefront. For the degeneration from the unstable conduction to the stable fast wave, the upstroke speed of the wavefront becomes faster and faster until reaching the steady-state conduction. For the degeneration from the unstable conduction to the stable slow wave, the upstroke speed of the wavefront becomes slower and slower until reaching the steady-state conduction. Because of the different upstroke speeds, the inactivation of I_{Na} is different. In Fig. 2 *B*, we plot the steady-state inactivation curve of I_{Na} (h_∞, *thick green*) and the trajectories of the three types of conduction in the V-h plane. The trajectory of the

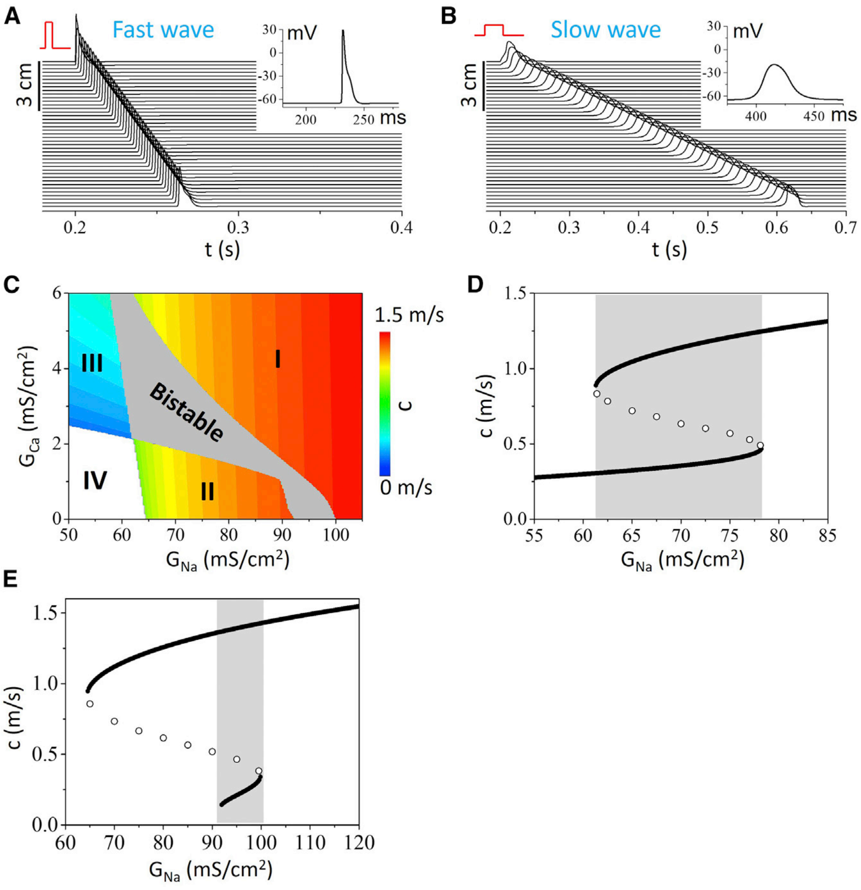

FIGURE 1 Bistable conduction in the cable equation of the HH model. (*A* and *B*) Stimulus-dependent fast (*A*) and slow (*B*) conduction in the same cable. The stimulus in (*A*) is 200 μA/cm^2 with a 0.5 ms duration while the stimulus in (*B*) is 5 μA/cm^2 with a 20 ms duration. The stimulus is applied to the first 0.225 cm of the cable. Insets show action potentials from the middle of the cable for the two cases. $G_{Na} = 95$ mS/cm^2 and $G_{Ca} = 0$ mS/cm^2. (*C*) Phase diagram showing conduction behaviors in the G_{Na} and G_{Ca} plane. Regions I and II are monostable fast conduction, region III is monostable slow conduction, region IV is conduction failure, and the gray region is bistable conduction. The phase diagram is obtained using the strong and weak stimulus protocols as in (*A* and *B*). (*D*) CV (c) versus G_{Na} for $G_{Ca} = 3$ mS/cm^2. Solid circles are stable conduction and open circles are unstable conduction (the saddle points). The saddle points are determined by a stimulus very close to the critical stimulus (see Fig. 2 *A* for an example). The gray marks the bistable region. (*E*) Same as (*D*) but for $G_{Ca} = 0$. To see this figure in color, go online.

steady-state slow wave (*thick red*) undergoes a path that is very close to the steady-state inactivation curve, while the trajectory of the steady-state fast wave (*thick blue*) undergoes a path that is far away from the steady-state curve. The thin red and blue trajectories are the transient ones degenerating from the steady-state unstable wave (*dashed*) to the two stable waves, respectively. In other words, the steady-state unstable trajectory (*dashed*) is the separatrix of the two stable waves.

As indicated by the two cases above, a slower upstroke speed causes more I_{Na} inactivation, which in turn results in an even slower upstroke in the next conduction site, or vice versa, forming a positive feedback loop. Therefore, the formation of the stable fast and slow conduction in the cable is a result of the positive feedback in upstroke speed of the wavefront mediated by I_{Na} inactivation, result in two modes of I_{Na} activation/inactivation. This causes bistable conduction to occur in the intermediate range of G_{Na}. When G_{Na} is too large, the high I_{Na} mode is always activated and thus only the fast wave occurs. When G_{Na} is too small, the low I_{Na} is not large enough to support the slow wave and thus only the high I_{Na}-mediated fast wave is observed. However, I_{Ca} can rescue the slow wave and thus extends the bistable conduction in a wider parameter space (Fig. 1 *C*).

To further understand the mechanism of bistable conduction, we obtain an analytical solution using a two-variable model, which includes only I_{Na} and I_L, i.e.,

$$\frac{\partial V}{\partial t} = \frac{\partial^2 V}{\partial x^2} + \sigma_{Na} m_\infty(V) h - G_L(V - E_L) \tag{8}$$

$$\frac{\partial h}{\partial t} = \frac{h_\infty(V) - h}{\tau_h}, \tag{9}$$

where V is voltage and h the inactivation gating variable of I_{Na}. σ_{Na} is a parameter proportional to G_{Na}. $m_\infty(V)$ and $h_\infty(V)$ are Heaviside functions:

$$m_\infty(V) = \begin{cases} 0, & V < V_m \\ 1, & V \geq V_m \end{cases} \tag{10}$$

and

$$h_\infty(V) = \begin{cases} 1, & V < V_h \\ h_0, & V \geq V_h \end{cases}. \tag{11}$$

By using the moving coordinate system and continuation conditions, one can obtain that the CV (c) satisfies the

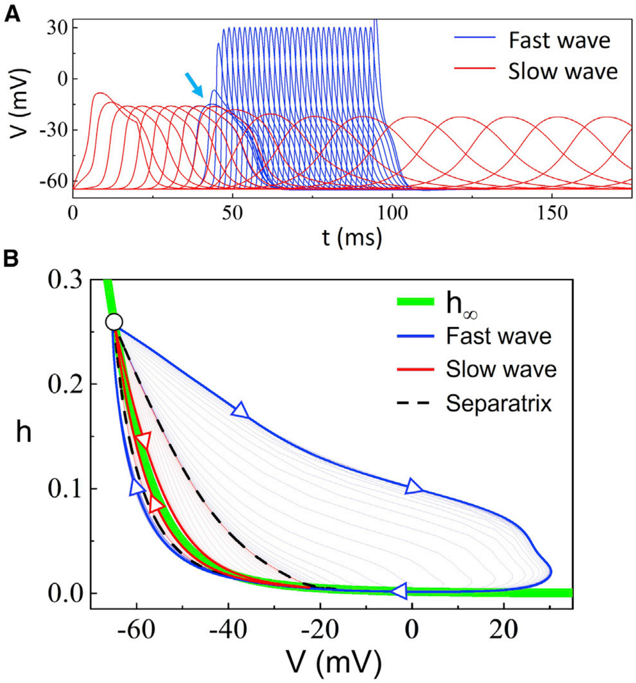

FIGURE 2 Mechanism of I_{Na}-mediated bistable conduction. (*A*) Formation of stable fast (*blue*) and slow (*red*) conduction from the unstable conduction in the absence of I_{Ca}. The fast wave is induced by a stimulus (I_{stim} = 22.32155910275 μA/cm^2 with 20 ms in duration) slightly above the critical strength, and the slow wave by a stimulus (I_{stim} = 22.32155910270 μA/cm^2 with 20 ms in duration) slightly below the critical strength. Voltage traces are recorded at sites 0.225 cm apart along the cable. The blue and red traces are overlapped until a distance away (marked by the *arrow*) from the stimulation site. G_{Na} = 92 mS/cm^2 and G_{Ca} = 0. (*B*) Na^+ channel inactivation properties during the fast and slow waves. The thick green line is the steady-state inactivation curve of the Na^+ channel (h_∞). The thick red curve with arrows is the trajectory of the steady-state slow wave and the thin red curves are the ones recorded from the transition period from the unstable conduction to the stable slow conduction. The thick blue curve with arrows is the trajectory of the steady-state fast wave and the thin blue curves are the ones recorded from the transition period from the unstable conduction to the stable fast conduction. The black dashed curve is the separatrix of the two types of waves, which is the trajectory taken from the unstable conduction marked in (*A*) before the arrow. To see this figure in color, go online.

following equation (see supporting material for detailed derivation):

$$-(V_m - E_L)\sqrt{c^2+4G_L} + \sigma_{Na}\left[\left(\beta + \frac{h_0}{G_L}\right)\delta - \frac{\beta}{\tau_h c}\right] = 0, \qquad (12)$$

where $\delta = \frac{-c+\sqrt{c^2+4G_L}}{2}$, $\beta = \frac{(1-h_0)e^{\frac{-\varepsilon}{\tau_h c}}}{G_L - \frac{1}{(\tau_h c)^2} - \frac{1}{\tau_h}}$, and $\varepsilon = \frac{\ln\left(\frac{V_h - E_L}{V_m - E_L}\right)}{-c-\delta}$. One can rewrite Eq. 12 in the form as $\sigma_{Na} = f(c, \tau_h, V_m, V_h, h_0, G_L, E_L)$, i.e., σ_{Na} is expressed as a nonlinear function of c. Fig. 3 *A* plots c versus σ_{Na} calculated using this formulation with other parameters fixed, showing that c is bistable in a certain range of σ_{Na}. One can also numerically solve Eq. 12 to obtain c when the parameters are given. Fig. 3 *B* shows the conduction behaviors versus σ_{Na} and τ_h, showing that the bistable region decreases as τ_h increases. We perform additional simulations of the one-dimensional cable with the HH model to investigate the effects of I_{Na} kinetics on the conduction behaviors. Fig. 3 *C* shows the conduction behaviors versus τ_h and τ_m (activation time constant of I_{Na}) and Fig. 3 *D* shows the conduction behaviors versus G_{Na} and τ_h. Note that the phase diagram in Fig. 3 *D* is similar to that in Fig. 3 *B*, indicating that theoretical predictions agree well with the numerical simulation results of the HH model.

Robustness of bistable conduction

In the case of *Aglantha digitale* (13), based on their observation that the fast wave is mediated by I_{Na} and the slow wave by I_{Ca}, the authors hypothesized that to facilitate the fast and slow conduction in the same axon, the Na^+ channel activation threshold is much higher than the Ca^{2+} channel activation threshold so that during I_{Ca}-mediated conduction (the slow wave), the peak voltage remains low enough to avoid activation of I_{Na}. On the other hand, in the case of cockroach experiments (14), since the slow wave was still mediated by I_{Na}, the authors then hypothesized that there might exist another type of I_{Na} with a low activation threshold in the same axon to explain their observations. In other words, theses authors hypothesized that the fast and slow conduction are mediated separately by two inward currents with a certain required difference in activation thresholds (we call it dual-threshold hypothesis). In the original HH model, since there is only one inward current (i.e., I_{Na}), it can only exhibit a single-threshold response (Fig. 4 *A*). On the other hand, after adding I_{Ca} to the HH model, it can exhibit a dual-threshold response in which two types of action potentials occur depending on the stimulus strength (Fig. 4 *B*). Since there are two distinct types of action potentials, one would expect that each type will give rise to a conduction in the cable, resulting in stimulus-dependent fast and slow conduction. This raises a question: which of the two mechanisms is robust or more likely to occur in the real systems?

To address this question, we perform the following investigations. We first carry out a large number of simulations of the cable equation with randomly selected parameters in assigned intervals (see details in supporting material). We detect the parameter sets giving rise to both fast and slow conduction. We then use these parameter sets to perform simulations using the nonspatial single-point model (Eq. 5) to filter out the single-threshold and dual-threshold responses by examining the stimulus-response relationship as shown in Fig. 4, *A* and *B*. The parameter sets (*open dots*) exhibiting single-threshold responses are plotted in Fig. 4 *C* and those exhibiting dual-threshold responses in Fig. 4 *D*. The data points are plotted on the plane of the half-activation voltages for Na^+ channels

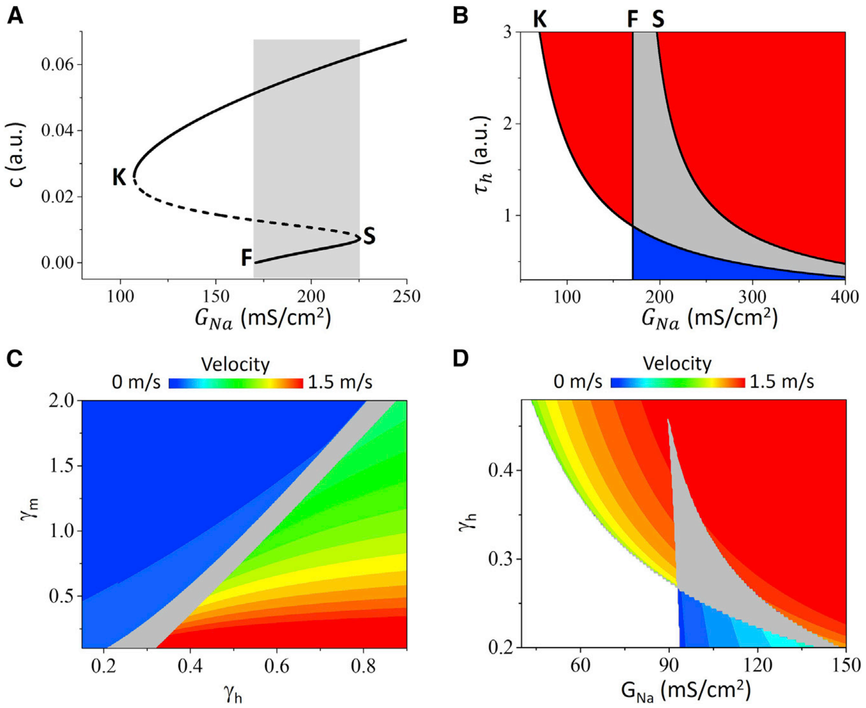

FIGURE 3 Effects of I_{Na} kinetics on bistable conduction. (*A*) Conduction velocity c versus G_{Na} calculated from the analytical result Eq. 12. K and S mark the saddle-node bifurcation points, and F marks the point of conduction failure of the slow conduction. Solid lines are stable conduction and dashed line is unstable conduction. Gray marks the bistable conduction region. $\tau_h = 1.6$, $V_m = -19.5$ mV, $V_h = -55.5$ mV, $h_0 = 0.075$, $G_L = 0.3$, $E_L = -65$ mV. We used $\sigma_{Na} = 2.1G_{Na}$ for Eq. 12. (*B*) Conduction behaviors versus G_{Na} and τ_h obtained from the analytical result Eq. 12. K, F, and S are the boundaries as marked on (*A*). The gray region is the bistable region. The red regions are monostable fast conduction, the blue region is monostable slow conduction, and the blank region is conduction failure. The parameters are the same as for (*A*). (*C*) Conduction behaviors versus γ_h and γ_m from the simulation of the cable equation using the HH model. The gray region is the bistable conduction region. $G_{Na} = 100$ mS/cm^2 and $G_{Ca} = 0$. (*D*) Conduction behaviors versus G_{Na} and γ_h from the simulation of the cable equation using the HH model. Other parameters are the same as for (*C*). The phase diagrams in (*C* and *D*) are obtained and colored the same way as for Fig. 1 *C*. To see this figure in color, go online.

($V_{Na,1/2}$) and Ca^{2+} channels ($V_{Ca,1/2}$). The definitions and ranges of $V_{Na,1/2}$ and $V_{Ca,1/2}$ in the model are detailed in supporting material. We also plot the experimentally measured $V_{Na,1/2}$ (*red squares*) and $V_{Ca,1/2}$ (*green circles*) surveyed from literature by Catterall et al. (20,21). $V_{Na,1/2}$ for the known 10 members of the Na^+ channel family ranges from −56 to −8.8 mV (20), and $V_{Ca,1/2}$ for the 10 members of the Ca^{2+} channel family ranges from −46 to 14 mV (21). $V_{Na,1/2}$ and $V_{Ca,1/2}$ are plotted in a way so that all possible combinations of the 10 members of the Na^+ channel family and the 10 members of the Ca^{2+} channel family fall inside the dashed red box. Therefore, we argue that the parameter sets fall inside the box are the ones that can occur in the real system.

The data sets exhibiting single-threshold response fall both inside and outside the dashed box (Fig. 4 *C*). We plot the distributions of CV for the fast and slow conduction in Fig. 4 *E*, and the ratios of the fast-to-slow CV in Fig. 3 *F*. The average slow CV is 0.23 ± 0.08 m/s and the average fast CV is 1.10 ± 0.32 m/s. The average of the fast-to-slow CV ratio is 7.0 ± 7.84. The fast-to-slow CV ratios were 5–10 in the experimentally measured CVs in the giant axons of *Aglantha digitale* (13) and cockroaches (14), which are in the same range as obtained in the simulations. Therefore, the bistable conduction mechanism agrees well with experimental data and is robust.

The data sets exhibiting dual-threshold response fall into two groups (Fig. 4 *D*). One group is completely outside the box (*lower right*), which occurs for $V_{Na,1/2}$ is 40 mV higher than $V_{Ca,1/2}$. The other group can still fall into the box (*upper left*), which occurs for $V_{Ca,1/2}$ is 30 mV higher than $V_{Na,1/2}$. However, some caveats of this group of data are worth noting. The slow wave is mediated by I_{Na} and the fast wave by I_{Ca}, which is not what occurs in either the jellyfish (13) or the cockroaches (14). Although the data sets were filtered through the dual-threshold criterion using the nonspatial single-point model (Eq. 5), when we check the behaviors in the cable, the Na^+ channel exhibit similar response as in Fig. 2, indicating that the mechanism may still be bistable conduction as in the single-threshold case. For example, when we remove I_{Ca} from the data sets in Fig. 4 *D*, we can still observe bistable conduction. To show this, we perform the same simulations using the parameter sets in Fig. 4 *D* without I_{Ca} (see Fig. S2 *A*), the parameter sets outside the box disappear, but about 50% of the parameter sets inside the box are retained. Another evidence to support this is that if we slow the Na^+ channel inactivation, this group of parameter sets will disappear. We perform the same simulations as in Fig. 4 except that the inactivation is slowed by increasing $\gamma_h = 0.35$ to $\gamma_h = 1$ (see Fig. S2 *B*). The upper group disappears, but the lower group is retained and still outside the dashed box. The results shown in Figs. 4 and S2 indicate that the dual-threshold mechanism is difficult to be satisfied in the real systems.

In the model, the resting potential is set at −65 mV. If we lower the resting potential, e.g., to −75 mV, we observe almost the same results (Fig. S3) as those shown in Fig. 4 and S2 except that the minimum I_{Na} or I_{Ca} activation threshold for bistable conduction has a roughly 10 mV shift due to the lowering of the resting potential.

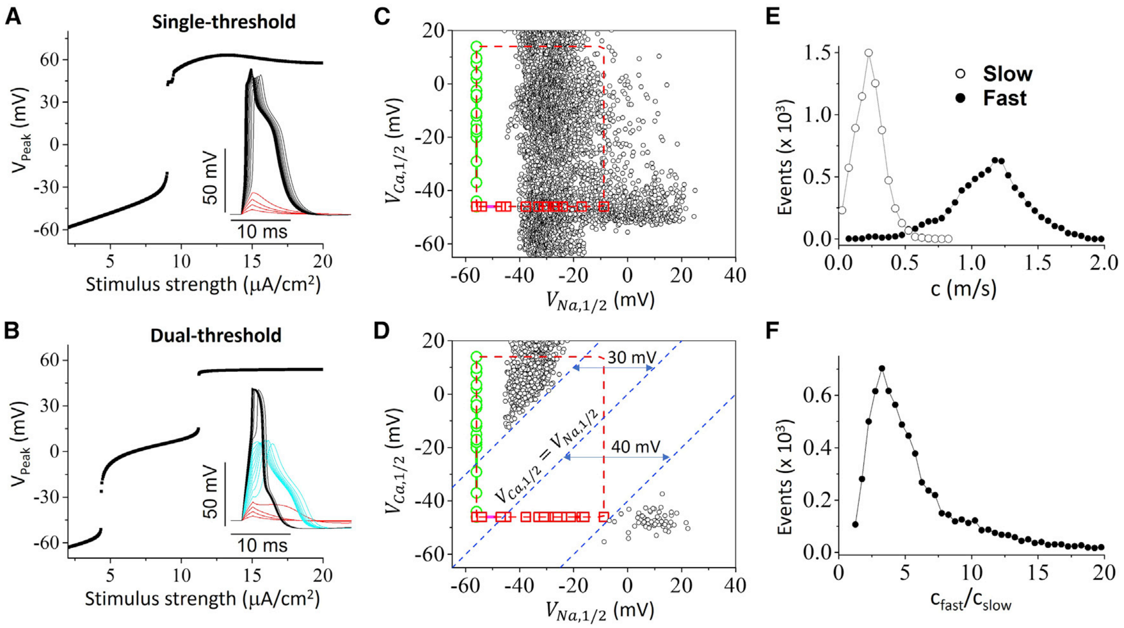

FIGURE 4 Robustness of bistable conduction. (*A*) Peak voltage versus stimulus strength for a case exhibiting a single threshold. Inset shows the voltage traces for different stimulus strengths. Red traces are the subthreshold responses. Simulations are carried out using the nonspatial single-point model (Eq. 5). (*B*) Same as (*A*) but for a case exhibiting dual thresholds. Inset shows that, besides the subthreshold response, there are two distinct types of action potentials. (*C*) Parameter sets (*black*) that give rise to bistable conduction filtered with the single-threshold criterion, plotted in the $V_{Na,1/2}$ and $V_{Ca,1/2}$ plane. Red squares are $V_{Na,1/2}$ taken from Catterall et al. (20) and green circles are $V_{Ca,1/2}$ taken from Catterall et al. (21). (*D*) Same as (*C*) but for the parameter sets filtered with the dual-threshold criterion. Dashed blue lines are reference lines marking the minimum difference of the two activation thresholds. (*E*) CV distributions for the slow and fast conduction calculated from the data shown in (*C*). (*F*) Distribution of the fast-to-slow CV ratio calculated from the same data in (*C*). To see this figure in color, go online.

DISCUSSION

Nerve conduction can be well described by the cable equation with the HH model (5–9). It is well known that the cable equation with FitzHug-Nagumo (FHN) model or the HH model exhibits monostable conduction (10). In this study, we show that the cable equation with the HH model can exhibit bistable conduction in which a fast and a slow stable conduction occur in the same cable depending on the stimulus strength. The bistable behavior is a result of the positive feedback of the wavefront upstroke speed mediated by the Na^+ channel inactivation properties. In other words, this positive feedback cause two stable modes of I_{Na} activation, which occurs when the Na^+ channel inactivation is relatively fast (see also Fig. 3). Unlike the fast conduction that occurs as long as G_{Na} is greater than a critical value, bistable conduction can only occur in an intermediate range of G_{Na}. This is because when G_{Na} is too large, the high I_{Na} mode is always activated and thus only the fast wave can occur. When G_{Na} is too small, the low I_{Na} cannot support a stable slow conduction, and thus only the fast conduction can occur. However, the failed slow conduction can be rescued by the addition of I_{Ca}, which can substantially extend the bistable conduction regime (see Fig. 1 *C*). We use an analytical treatment of the simplified cable model to demonstrate that bistable conduction can be mediated by a single inward current, namely I_{Na}. Using simulations with randomly drawing parameter sets, we also show that the bistable conduction is robust, which can occur well within the experimentally determined ranges of the activation thresholds of the known Na^+ and Ca^{2+} channel families.

Our computer simulation results agree well with the experimental observations of electrical conduction in the giant axons of jellyfish and cockroaches. For example, the action potential profiles in the fast and slow waves and the ratios of the fast-to-slow CV in the simulations agree with those shown in experiments in both jellyfish and cockroaches. More importantly, our theoretical study unifies the seemingly different experimental observations in jellyfish and cockroaches to the same general mechanism. In the jellyfish experiments, Mackie and Meech (13) showed that the slow wave was blocked by Ca^{2+} channel blockers, and thus concluded that the slow wave was mediated by I_{Ca}. This led them to hypothesize that the activation threshold of I_{Na} has to be much higher than that of I_{Ca} to allow the two waves to occur. As shown in our simulations (Fig. 1 *C*), although I_{Ca} is not required for bistable conduction, it potentiates bistable conduction by rescuing the slow conduction. Therefore, blocking it will suppress the slow conduction, agreeing with the experimental observation. On the other hand, in the cockroach experiments, Hochner and Spira (14) found that the slow wave was blocked not by Ca^{2+} channel blockers but by Na^+ channel blockers,

differing from the jellyfish experiments. They then hypothesized that a low-threshold I_{Na} must be responsible for the slow wave. Our simulations showed that I_{Ca} or another low-threshold I_{Na} is not required since a single I_{Na} could produce both the slow and fast conduction via the bistable conduction dynamics. In other words, the fast and slow conduction observed in cockroaches may originate from the same I_{Na}. A moderate I_{Na} reduction may block the slow wave but not the fast wave while a strong I_{Na} reduction can block both waves (see Fig. 1, *C* and *E*). Therefore, the experimental observations in both jellyfish and cockroaches can be explained by the same mechanism in different parameter regimes.

Besides the giant axons in jellyfish and cockroaches, bistable conduction may also occur in other nerve systems. For example, it was shown that the spontaneous waves and the evoked waves exhibit distinct CVs in the same rat visual cortex (15,16), which could be a result of bistable conduction. In addition, bimodal CV distributions were widely observed in sensory and motor nerves (22–28), which were traditionally attributed to the size difference of the fibers. However, bistable conduction can be a candidate mechanism for bimodal CV distributions, which needs to be verified in future experimental studies.

Note that, although the duration of action potential in the slow wave (~40 ms) shown in Fig. 1 *B* matches quantitatively well with the ones measured in experiments (Fig. S1 and Fig. 1 in Meech and Mackie (29)), but the action potential of the fast wave in Fig. 1 *A* has a duration of about 10 ms, which is much longer than the one recorded in the experiment (~2 ms, see Fig. S1). The main cause of action potential lengthening is due to the shift of the K^+ channel kinetics (see Table 1), which reduces activation of the K^+ current. The purpose of shifting the K^+ channel kinetics is to result in bistable conduction in the HH model. However, there are multiple K^+ currents in the giant axon of *Aglantha digitale*. In the experimental studies by Meech and Mackie (29,30), they showed that there are three kinetically distinct K^+ currents in the giant axon of *Aglantha digitale*, and these currents behave like the A-type current that is widely observed in neurons (31). If one adds an A-type current with a proper conductance into the cable model (Fig. S5), one can shorten the action potential to quantitatively match the ones recorded in experiments (compare the action potentials in Fig. S5 with the ones in Fig 1 in Meech and Mackie (29)), but still retains the bistable conduction behavior.

Different modes of conduction in the same cable have been shown in the HH model (5,32) as well as in the FHN model (10,33), i.e., for the same parameter set, there are a fast and a slow conduction. However, since the slow conduction is unstable and decremental, the two conduction modes are not a bistable behavior. A bistable behavior in the cable model with the FHN equations was demonstrated by Rinzel and Terman (34), in which two action potential repolarization behaviors were observed in the same cable during conduction. Namely, in one state, the action potential repolarizes normally but, in the other state, the action potential fails to repolarize. However, since in the two states, the upstrokes of wavefront are roughly the same, the conduction velocities do not exhibit two distinction values. In other words, it is a bistable behavior in repolarization not in depolarization, differing from the bistable conduction shown in this study. Note that the bistable conduction is mediated by fast Na^+ channel inactivation, which is absent in the FHN model, and thus the bistable conduction behavior cannot occur in the FHN-type models.

Finally, as shown in our simulations (Fig. 4), the dual-threshold mechanism is theoretically plausible; however, it requires a very large minimum difference in the activation thresholds of I_{Na} and I_{Ca}. Meech and Mackie (29,35) showed that the slow conduction is mediated by the T-type Ca^{2+} current and fast conduction by the Na^+ current, with the Na^+ current activation threshold being much higher than that of the Ca^{2+} current. However, using the data of Na^+ and Ca^{2+} channel activation threshold surveyed by Catterall et al. (20,21), we show that this mechanism is much less robust, i.e., the condition for the mechanism cannot be easily satisfied in the real systems. On the other hand, the bistable conduction mechanism can be easily achieved without requiring any gap or correlation between the activation thresholds of the two types of ionic currents. Moreover, the bistable conduction mechanism may also have an evolutionary advantage over the dual-threshold mechanism since a single rather than two inward currents can provide two survival functions as in the jellyfish. Although it is unclear what are the functional roles of the fast and slow conduction in other species or diseases, as it is well known that bistability is a ubiquitous phenomenon in biology and responsible for many biological functions (36–39), we believe that bistable conduction may also play important roles in nerve functions under healthy and diseased conditions. As shown in our study, bistable conduction occurs in the cable equation with the HH model with modifications, we believe that it is a generic mechanism that is applicable to not only the nerve systems but also other electrically excitable tissue, such as cardiac muscles.

SUPPORTING MATERIAL

Supporting material can be found online at https://doi.org/10.1016/j.bpj.2022.08.006.

AUTHOR CONTRIBUTIONS

Z.Q. conceived the project, supervised the research, provided funding, and wrote the manuscript. Z.Z. performed the simulations and mathematical analyses. Z.Q. and Z.Z. analyzed the results and edited the manuscript.

ACKNOWLEDGMENTS

This study was supported by National Institutes of Health grants R01 HL134709 and R01 HL139829.

DECLARATION OF INTERESTS

The authors declare no competing interests.

REFERENCES

1. Castelfranco, A. M., and D. K. Hartline. 2016. Evolution of rapid nerve conduction. *Brain Res.* 1641:11–33.
2. Hursh, J. B. 1939. Conduction velocity and diameter of nerve fibers. *American Journal of Physiology-Legacy Content.* 127:131–139.
3. Waxman, S. G. 1980. Determinants of conduction velocity in myelinated nerve fibers. *Muscle Nerve.* 3:141–150.
4. Rosenthal, J. J., and F. Bezanilla. 2000. Seasonal variation in conduction velocity of action potentials in squid giant axons. *Biol. Bull.* 199:135–143.
5. Cooley, J. W., and F. A. Dodge. 1966. Digital computer solutions for excitation and propagation of the nerve impulse. *Biophys. J.* 6:583–599.
6. Goldman, L., and J. S. Albus. 1968. Computation of impulse conduction in myelinated fibers; theoretical basis of the velocity-diameter relation. *Biophys. J.* 8:596–607.
7. Parnas, I., and I. Segev. 1979. A mathematical model for conduction of action potentials along bifurcating axons. *J. Physiol. (Camb.).* 295:323–343.
8. Miller, R. N., and J. Rinzel. 1981. The dependence of impulse propagation speed on firing frequency, dispersion, for the Hodgkin-Huxley model. *Biophys. J.* 34:227–259.
9. Young, R. G., A. M. Castelfranco, and D. K. Hartline. 2013. The "Lillie transition": models of the onset of saltatory conduction in myelinating axons. *J. Comput. Neurosci.* 34:533–546.
10. Keener, J. P., and J. Sneyd. 1998. Mathematical Physiology. Springer, New York.
11. Satterlie, R. 2018. Jellyfish locomotion. *In* Oxford Research Encyclopedia, Neuroscience (oxfordre.Com/neuroscience). Oxford University Press.
12. Meech, R. W. 2019. Electrophysiology and behavior of Cnidarian nervous systems. *In* Oxford Research Encyclopedia, Neuroscience (oxfordre.Com/neuroscience). Oxford University Press.
13. Mackie, G. O., and R. W. Meech. 1985. Separate sodium and calcium spikes in the same axon. *Nature.* 313:791–793.
14. Hochner, B., and M. E. Spira. 1986. Two distinct propagating regenerative potentials in a single ethanol-treated axon. *Brain Res.* 398:164–168.
15. Xu, W., X. Huang, …, J.-Y. Wu. 2007. Compression and reflection of visually evoked cortical waves. *Neuron.* 55:119–129.
16. Han, F., N. Caporale, and Y. Dan. 2008. Reverberation of recent visual experience in spontaneous cortical waves. *Neuron.* 60:321–327.
17. Hodgkin, A. L., and A. F. Huxley. 1952. A quantitative description of membrane current and its application to conduction and excitation in nerve. *J. Physiol. (Camb.).* 117:500–544.
18. Medlock, L., L. Shute, …, A. V. Ferguson. 2018. Ionic mechanisms underlying tonic and burst firing behavior in subfornical organ neurons: a combined experimental and modeling study. *J. Neurophysiol.* 120:2269–2281.
19. Rush, S., and H. Larsen. 1978. A practical algorithm for solving dynamic membrane equations. *IEEE Trans. Biomed. Eng.* 25:389–392.
20. Catterall, W. A., A. L. Goldin, and S. G. Waxman. 2005. International union of pharmacology. XLVII. Nomenclature and structure-function relationships of voltage-gated sodium channels. *Pharmacol. Rev.* 57:397–409.
21. Catterall, W. A., E. Perez-Reyes, …, J. Striessnig. 2005. International union of pharmacology. XLVIII. Nomenclature and structure-function relationships of voltage-gated calcium channels. *Pharmacol. Rev.* 57:411–425.
22. Andrew, B. L., and N. J. Part. 1972. Properties of fast and slow motor units in hind limb and tail muscles of the rat. *Q. J. Exp. Physiol. Cogn. Med. Sci.* 57:213–225.
23. Calvin, W. H., and G. W. Sypert. 1976. Fast and slow pyramidal tract neurons: an intracellular analysis of their contrasting repetitive firing properties in the cat. *J. Neurophysiol.* 39:420–434.
24. Cummins, K. L., L. J. Dorfman, and D. H. Perkel. 1979. Nerve fiber conduction-velocity distributions. II. Estimation based on two compound action potentials. *Electroencephalogr. Clin. Neurophysiol.* 46:647–658.
25. Milner, T. E., and R. B. Stein. 1981. The effects of axotomy on the conduction of action potentials in peripheral sensory and motor nerve fibres. *J. Neurol. Neurosurg. Psychiatry.* 44:485–496.
26. Todnem, K., G. Knudsen, …, J. A. Aarli. 1989. The non-linear relationship between nerve conduction velocity and skin temperature. *J. Neurol. Neurosurg. Psychiatry.* 52:497–501.
27. van Veen, B. K., R. L. Schellens, …, A. A. W. M. Gabreëls-Festen. 1995. Conduction velocity distributions compared to fiber size distributions in normal human sural nerve. *Muscle Nerve.* 18:1121–1127.
28. Ni, Z., F. Vial, …, M. Hallett. 2020. Measuring conduction velocity distributions in peripheral nerves using neurophysiological techniques. *Clin. Neurophysiol.* 131:1581–1588.
29. Meech, R. W., and G. O. Mackie. 1993. Ionic currents in giant motor axons of the jellyfish, Aglantha digitale. *J. Neurophysiol.* 69:884–893.
30. Meech, R. W., and G. O. Mackie. 1993. Potassium channel family in giant motor axons of Aglantha digitale. *J. Neurophysiol.* 69:894–901.
31. Zemel, B. M., D. M. Ritter, …, T. Muqeem. 2018. A-type KV channels in dorsal root ganglion neurons: diversity, function, and dysfunction. *Front. Mol. Neurosci.* 11:253.
32. Huxley, A. F. 1959. Ion movements during nerve activity. *Ann. N. Y. Acad. Sci.* 81:221–246.
33. Rinzel, J., and J. B. Keller. 1973. Traveling wave solutions of a nerve conduction equation. *Biophys. J.* 13:1313–1337.
34. Rinzel, J., and D. Terman. 1982. Propagation phenomena in a bistable reaction-diffusion system. *SIAM J. Appl. Math.* 42:1111–1137.
35. Meech, R. W., and G. O. Mackie. 1995. Synaptic potentials and threshold currents underlying spike production in motor giant axons of Aglantha digitale. *J. Neurophysiol.* 74:1662–1670.
36. Gardner, T. S., C. R. Cantor, and J. J. Collins. 2000. Construction of a genetic toggle switch in Escherichia coli. *Nature.* 403:339–342.
37. Sha, W., J. Moore, …, J. C. Sible. 2003. Hysteresis drives cell-cycle transitions in Xenopus laevis egg extracts. *Proc. Natl. Acad. Sci. USA.* 100:975–980.
38. Xiong, W., and J. E. Ferrell, Jr. 2003. A positive-feedback-based bistable 'memory module' that governs a cell fate decision. *Nature.* 426:460–465.
39. Qu, Z., G. Hu, …, J. N. Weiss. 2014. Nonlinear and stochastic dynamics in the heart. *Phys. Rep.* 543:61–162.

Biophysical Journal, Volume *121*

Supplemental information

Bistable nerve conduction

Zhaoyang Zhang and Zhilin Qu

Supplemental Information

A. Supplemental Methods

1. Assigned intervals for random parameter drawing

To evaluate the robustness of the two mechanisms, we carried out a large number of simulations with randomly drawn parameter sets. In these simulations, besides drawing the maximum conductance and time constants randomly, we also randomly shifted the kinetics of the ionic currents to investigate the effects of their activation thresholds on conduction. To shift the I_{Na} kinetics, we replaced V for α_m, β_m, α_h, and β_h by ($V+V_{Na,shift}$). In the control, the half-activation potential for I_{Na} is $V_{Na,1/2} \approx -35\ mV$ (at which m_∞=0.5). After the shift, the half-activation potential becomes,

$$V_{Na,1/2} = -35 - V_{Na,shift} \qquad \text{(S1)}$$

Similarly, we shifted the I_{Ca} kinetics by replacing V for d_∞ and f_∞ with ($V+V_{Ca,shift}$). The half-activation voltage for I_{Ca} at control is $V_{Ca,1/2} = -14\ mV$ (at which d_∞=0.5), and after the shift, it becomes,

$$V_{Ca,1/2} = -14 - V_{Ca,shift} \qquad \text{(S2)}$$

We also shifted the I_K kinetics by replacing V for α_n and β_n with ($V+V_{K,shift}$).

The randomly drawn parameter sets used for the simulations for Fig.3, Fig.4, and Fig.S2 were uniformly drawn from the following pre-assigned intervals: $G_{Na}\in[50,200]$, $G_K\in[20,60]$, $G_{Ca}\in[0,10]$, $V_{Na,shift}\in[-75,25]$, $V_{Ca,shift}\in[-50,50]$, $V_{K,shift}\in[-40,60]$, $\gamma_m\in[0.1,0.5]$, $\gamma_d\in[1,8]$, $\gamma_n\in[0.5,10]$, $k_f\in[8,2]$. The fixed parameters were the same as for control. Therefore, based on Eqs.S1 and S2 and the assigned intervals for $V_{Na,shift}$ and $V_{Ca,shift}$, the range for $V_{Na,1/2}$ is [-60, 40] mV and that for $V_{Ca,1/2}$ is [-64, 36] mV.

2. Theoretical model and analysis of bistable conduction

To understand the mechanism of bistable conduction mediated by Na^+ channel, we investigated the following simple cable equation:

$$\frac{\partial V}{\partial t} = \frac{\partial^2 V}{\partial x^2} + \sigma_{Na} m_\infty(V) h - G_L(V - E_L) \qquad \text{(S3)}$$

$$\frac{\partial h}{\partial t} = \frac{h_\infty(V) - h}{\tau_h} \qquad \text{(S4)}$$

For simplicity and analytical treatment, we set the diffusion constant D=1, and set $\sigma_{Na} = G_{Na}(E_{Na} - V_C)$ to be single parameter. We also assume τ_h is a constant independent of V. $m_\infty(V)$ and $h_\infty(V)$ are Heaviside functions of V as follows:

$$m_\infty(V) = \begin{cases} 0, & V < V_m \\ 1, & V \geq V_m \end{cases} \qquad \text{(S5)}$$

and

$$h_\infty(V) = \begin{cases} 1, & V < V_h \\ h_0, & V \geq V_h \end{cases} \qquad \text{(S6)}$$

We seek a travelling wave solution $V(x,t)$ with speed c in an infinite spatial domain: $x \in (-\infty, +\infty)$, which becomes a stationary front in the moving coordinate system (see Fig.S4) $\varepsilon = x - ct$, i.e.,

$$V(x,t) = U(x-ct) = U(\varepsilon) \quad \text{(S7)}$$

By substituting Eq.S7 into Eq.S3 and Eq.S4, one obtains the follow set of differential equations:

$$0 = U_{\varepsilon\varepsilon} + cU_\varepsilon + \sigma_{Na} m_\infty h - G_L(U - E_L) \quad \text{(S8)}$$

$$h_\varepsilon = \frac{h_\infty(U) - h}{-c\tau_h} \quad \text{(S9)}$$

in which $U_\varepsilon = \frac{dU}{d\varepsilon}$ and $U_{\varepsilon\varepsilon} = \frac{dU_\varepsilon}{d\varepsilon}$. The solution U(ε) satisfies the following boundary conditions: 1) $U_\varepsilon = U_{\varepsilon\varepsilon} = 0$ at $\varepsilon \to \pm\infty$; and 2) We assume that $m_\infty(V)$ changes from 1 to 0 at $\varepsilon = 0$, then $U(0) = V_m$ and $U_\varepsilon|_{\varepsilon=0^-} = U_\varepsilon|_{\varepsilon=0^+}$. Therefore, at the boundaries, U(ε) satisfies:

$$U(\varepsilon) = \begin{cases} U_0, & \varepsilon \to -\infty \\ V_m, & \varepsilon = 0 \\ E_L, & \varepsilon \to +\infty \end{cases} \quad \text{(S10)}$$

where $U_0 = \frac{\sigma_{Na} h_0 + G_L E_L}{G_L}$. Now the problem is to solve the linear differential equations in the subdomains of $\varepsilon < 0$ and $\varepsilon > 0$.

(i) $\varepsilon > 0$

Since $m_\infty = 0$ when $\varepsilon > 0$, then Eq.S8 becomes:

$$0 = U_{\varepsilon\varepsilon} + cU_\varepsilon - G_L(U - E_L) \quad \text{(S11)}$$

which has the following form of solution:

$$U(\varepsilon) = Ae^{\lambda_- \varepsilon} + E_L \quad \text{(S12)}$$

where $\lambda_- = \frac{-c - \sqrt{c^2 + 4G_L}}{2}$ is a solution of the characteristic equation $\lambda^2 + c\lambda - G_L(U - E_L) = 0$. Since $\lambda_- < 0$, $U(\varepsilon) = E_L$ at $\varepsilon \to +\infty$ is satisfied. Since $U(\varepsilon) = V_m$ at $\varepsilon = 0$, one obtains $A = V_m - E_L$. The solution of Eq.S9 in the whole spatial domain is:

$$h(\varepsilon) = \begin{cases} h_0 + (1 - h_0)e^{\frac{\varepsilon - \varepsilon_0}{c\tau_h}}, & \varepsilon < \varepsilon_0 \\ 1, & \varepsilon \geq \varepsilon_0 \end{cases} \quad \text{(S13)}$$

in which ε_0 is determined by $(V_m - E_L)e^{\lambda_- \varepsilon_0} + E_L = V_h$, which leads to

$$\varepsilon_0 = \frac{\ln\left(\frac{V_h - E_L}{V_m - E_L}\right)}{\lambda_-} \quad \text{(S14)}$$

(ii) $\varepsilon < 0$

Since $m_\infty = 1$ when $\varepsilon < 0$, using Eq.S13 for h, then Eq.S8 becomes

$$0 = U_{\varepsilon\varepsilon} + cU_\varepsilon + \sigma_{Na}[h_0 + (1 - h_0)e^{\frac{\varepsilon - \varepsilon_0}{c\tau_h}}] - G_L(U - E_L) \quad \text{(S15)}$$

Eq.S15 exhibits the following form of solution:

$$U(\varepsilon) = U_0 + Be^{\lambda_+ \varepsilon} + \beta e^{\frac{\varepsilon}{c\tau_h}} \qquad \text{(S16)}$$

where $\lambda_+ = \frac{-c+\sqrt{c^2+4G_L}}{2}$ is a solution of the characteristic equation $\lambda^2 + c\lambda - G_L(U - E_L) = 0$. Since $\lambda_+ > 0$, $U(\varepsilon) = U_0$ at $\varepsilon \to -\infty$ is satisfied. Since $U(\varepsilon) = V_m$ at $\varepsilon = 0$, one obtains $B = V_m - \beta - U_0$. Inserting Eq.S16 into Eq.S15, one obtains the following equation for β:

$$\frac{\beta}{(c\tau_h)^2} + \frac{c\beta}{c\tau_h} + \sigma_{Na}(1 - h_0)e^{\frac{-\varepsilon_0}{c\tau_h}} - \beta G_L = 0 \qquad \text{(S17)}$$

which gives rise to $\beta = \frac{\sigma_{Na}(1-h_0)e^{\frac{-\varepsilon_0}{c\tau_h}}}{G_L - \frac{1}{(c\tau_h)^2} - \frac{1}{\tau_h}}$.

Finally, applying the constrain that U is smooth at $\varepsilon = 0$, i.e., $U_\varepsilon|_{\varepsilon=0^-} = U_\varepsilon|_{\varepsilon=0^+}$, one has from Eq.S12 and Eq.S16 the following relation:

$$A\lambda_- = B\lambda_+ + \frac{\beta}{c\tau_h} \qquad \text{(S18)}$$

which leads to

$$-(V_m - E_L)\sqrt{c^2 + 4G_L} + \sigma_{Na}[(\beta + \frac{h_0}{G_L})\lambda_+ - \frac{\beta}{\tau_h c}] = 0 \qquad \text{(S19)}$$

Therefore, once the parameters are given, one can solve Eq.S19 to obtain the conduction speed c as a function of anyone of the parameters, which is discussed in the main text. Fig.S4 plots the two stable and the unstable traveling wave solutions of Eq.S12 and Eq.S16 with the c values calculated from Eq.S19.

B. Supplemental Figures

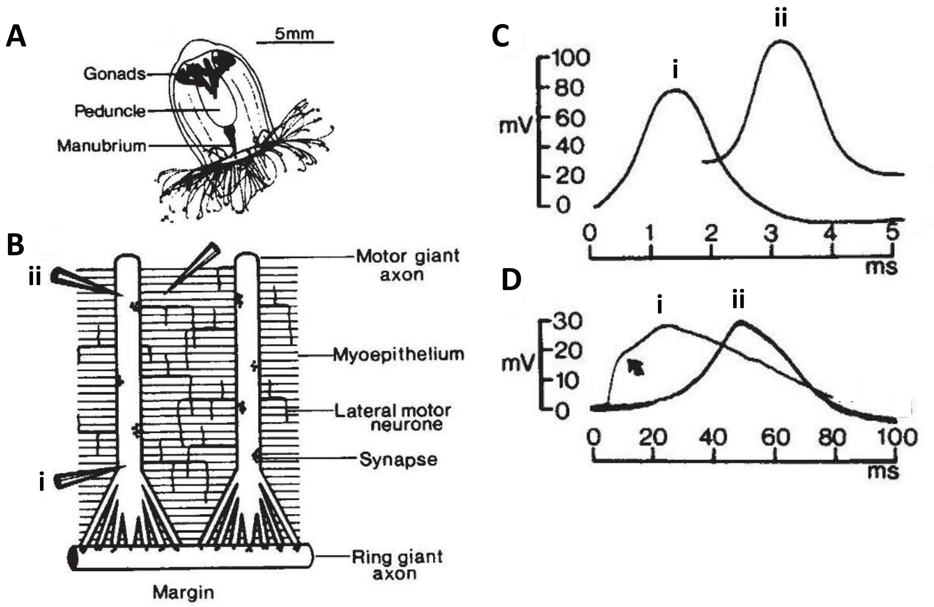

Figure S1. Action potential conduction in giant axon of *Aglantha digitale*. A. Schematic drawing of *Aglantha digitale.* **B**. Recording sites (i and ii) in the giant axon. **C**. Recorded action potentials during an escape swim, which are I_{Na}-mediated high amplitude and short duration excitations. **D**. Recorded action potentials during slow swim, which are I_{Ca}-mediated low amplitude and long duration excitations. This figure is modified from Mackie and Meech (1), see the original paper for a detailed description.

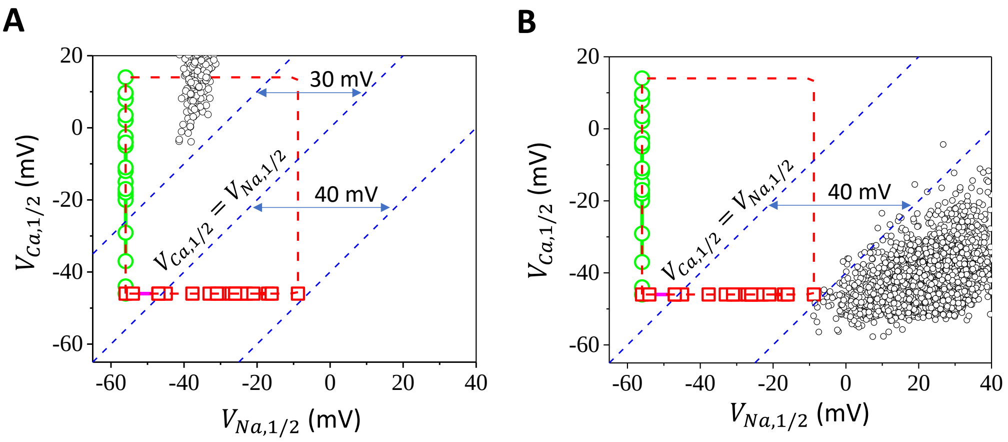

Figure S2. **Robustness of bistable conduction. A**. Parameter sets (black) exhibiting stimulus-dependent fast and slow conduction after I_{Ca} was removed from the model using the parameter sets in Fig.4D. The data points in the lower-right group are gone but there are data points in the upper-left group. This indicates that the upper-left group in Fig.4D may still undergo the bistable conduction even though the parameter sets were filtered using the dual-threshold criterion. **B**. Parameter sets (black) exhibiting stimulus-dependent fast and slow conduction after changing γ_h=0.35 to γ_h=1. Unlike Figs. 4 C or D, the data points in this panel were not filtered with the threshold criterion.

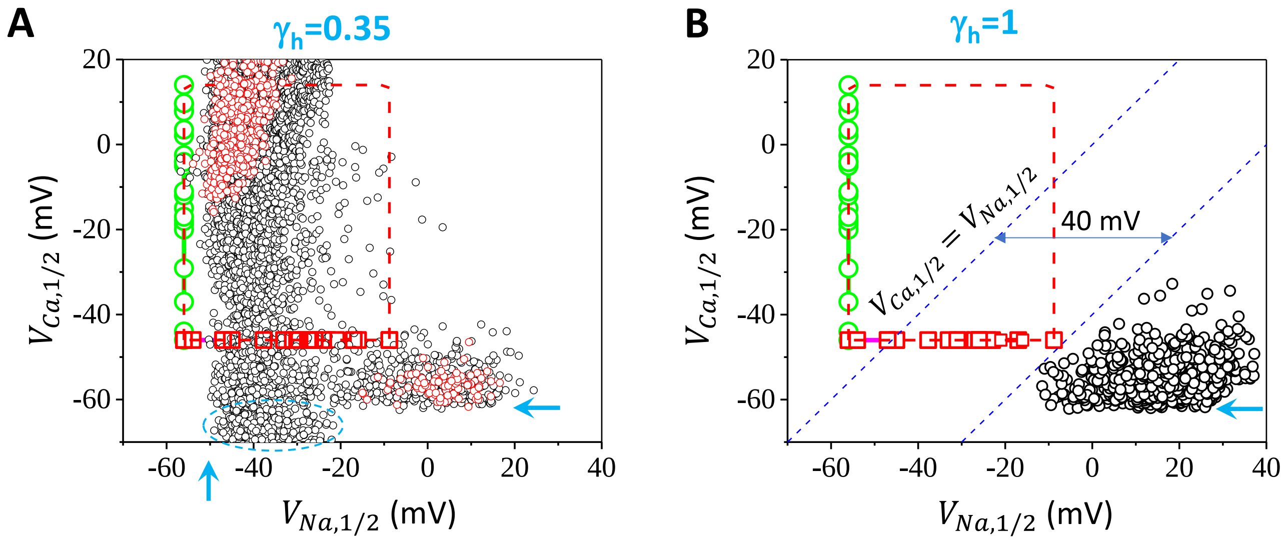

Figure S3. **Effects of lowering the resting potential**. Data sets exhibiting fast and slow conduction after changing the resting potential from -65 mV to -75 mV by changing E_L=-65 mV to E_L=-75 mV. **A**. γ_h=0.35. Red dots are the parameter sets filtered with the dual-threshold criterion. Black dots are the parameter sets filtered with the single-threshold criterion. **B**. γ_h=1. Simulation protocols and parameters are the same as for Fig.4 in the main text except the mentioned changes. The lowering of the resting potential caused two changes: down-shift of the $V_{Ca,1/2}$ boundaries (marked by horizontal arrows, ~10 mV lower comparing to Fig.4) and left-shift of the $V_{Na,1/2}$ boundary in A (marked by the vertical arrow, ~10 mV left-shifted comparing to Fig.4C). In other words, shifting the resting from -65 mV to -75 mV results in a ~10 mV shift in these boundaries. Beyond these boundaries, spontaneous oscillations occur and thus no conduction in the cable due to the low activation thresholds. The data points in the region marked by the dashed circle are the bistable conduction caused by I_{Na} alone in the absence of or under weak I_{Ca} so that the lower boundary becomes lower than the oscillation boundary caused by I_{Ca}.

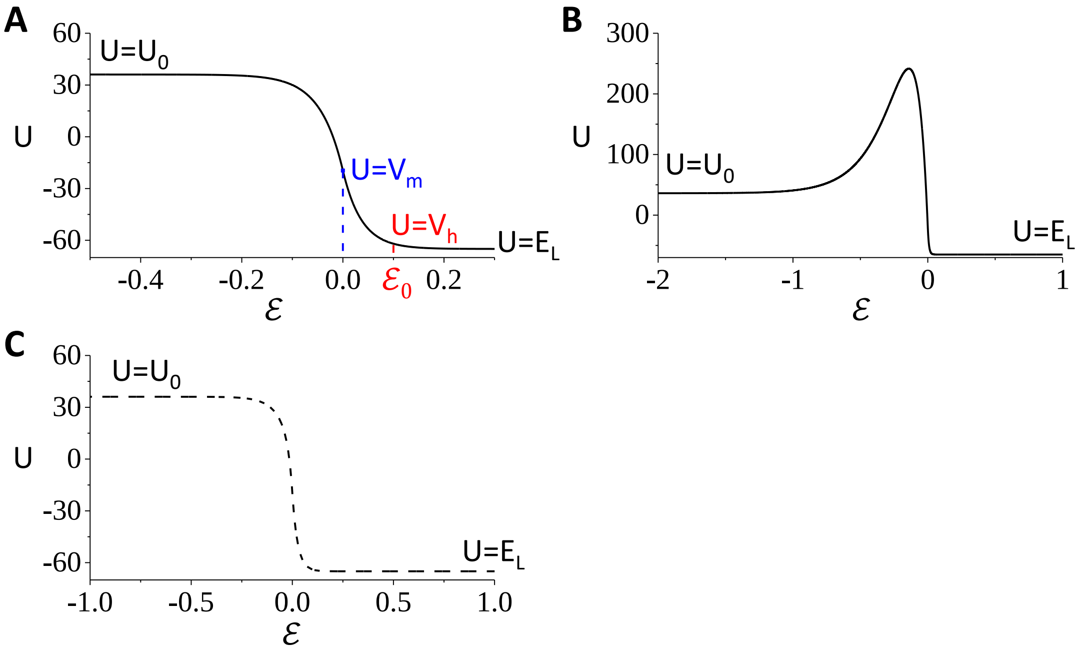

Figure S4. **Stable and unstable wavefronts in the moving coordinate from the simplified model**. **A**. The stable slow wave front of a bistable conduction. U=V_m when ε=0, which gives rise to m_∞=1 when ε<0 and m_∞=0 when ε>0. U=V_h when ε=ε_0, which gives rise to h_∞=h_0 when ε<ε_0 and h_∞=1 when ε>ε_0. **B**. The stable fast wave front of the same bistable conduction as in A. **C**. The unstable wave front of the same bistable conduction as in A and B. The traveling wave solutions in A-C were plots of Eq.S12 and Eq.S16 using c values calculated in Eq.S19 with the following parameter set: $\tau_h = 1.6$, $V_m = -19.5$ mV, $V_h = -55.5$ mV, $h_0 = 0.075$, $G_L = 0.3$, $E_L = -65$ mV. $G_{Na} = 190$ mS/cm^2. $\sigma_{Na} = 2.1G_{Na}$.

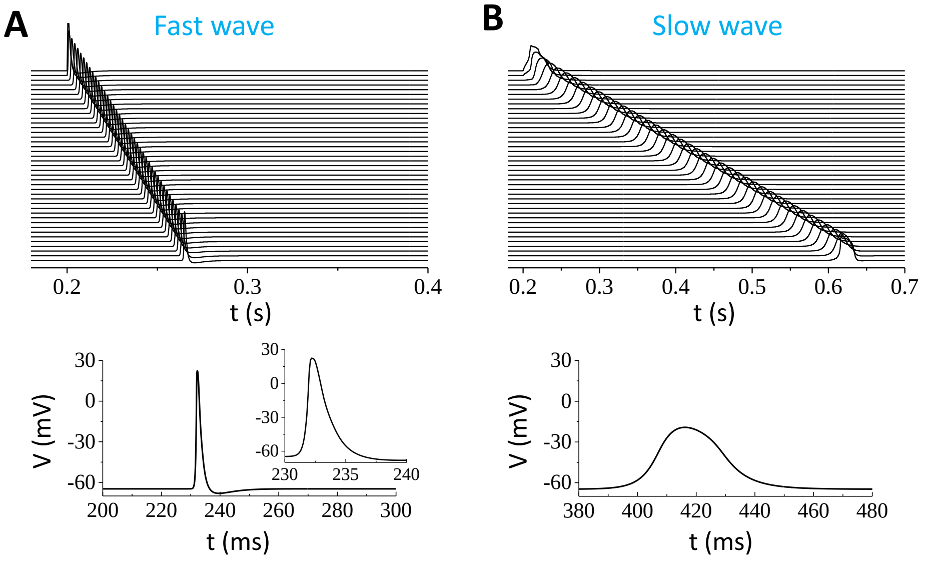

Figure S5. **Bistable conduction in the presence of A-type current**. Simulations are carried out the same way as for Figs.1 A and B in the main text except that an A-type current is added to the model. The formulation of I_A is taken from Medlock et al (2) with the following parameter changes: $p = 1$, $\Phi_m = -10$, $k_m = 1$ $\Phi_h = -30$, and $\tau_m = 3$.

References

1. Mackie, G. O., and R. W. Meech. 1985. Separate sodium and calcium spikes in the same axon. Nature 313(6005):791-793.
2. Medlock, L., L. Shute, M. Fry, D. Standage, and A. V. Ferguson. 2018. Ionic mechanisms underlying tonic and burst firing behavior in subfornical organ neurons: a combined experimental and modeling study. J Neurophysiol 120(5):2269-2281.
